# Comparison of Regularized Reconstruction and Ordered Subset Expectation Maximization Reconstruction in the Diagnostics of Prostate Cancer Using Digital Time-of-Flight ^68^Ga-PSMA-11 PET/CT Imaging

**DOI:** 10.3390/diagnostics11040630

**Published:** 2021-03-31

**Authors:** Olof Jonmarker, Rimma Axelsson, Ted Nilsson, Stefan Gabrielson

**Affiliations:** 1 CLINTEC, Department of Radiology, Karolinska Institutet, 171 77 Stockholm, Sweden; Rimma.Axelsson@ki.se (R.A.); stefan.gabrielson@ki.se (S.G.); 2 ME Radiology Huddinge, Karolinska University Hospital, 141 86 Stockholm, Sweden; 3 ME Medical Radiation Physics and Nuclear Medicine, Karolinska University Hospital, 141 86 Stockholm, Sweden; Ted.nilsson@sll.se; 4 Radiology Services, Christchurch Hospital, CDHB, P.O. Box 1600, Christchurch 8140, New Zealand

**Keywords:** prostate cancer, PSMA-PET/CT, reconstruction algorithms

## Abstract

In prostate cancer, the early detection of distant spread has been shown to be of importance. Prostate-specific membrane antigen (PSMA)-binding radionuclides in positron emission tomography (PET) is a promising method for precise disease staging. PET diagnostics depend on image reconstruction techniques, and ordered subset expectation maximization (OSEM) is the established standard. Block sequential regularized expectation maximization (BSREM) is a more recent reconstruction algorithm and may produce fewer equivocal findings and better lesion detection. Methods: ^68^Ga PSMA-11 PET/CT scans of patients with de novo or suspected recurrent prostate cancer were retrospectively reformatted using both the OSEM and BSREM algorithms. The lesions were counted and categorized by three radiologists. The intra-class correlation (ICC) and Cohen’s kappa for the inter-rater reliability were calculated. Results: Sixty-one patients were reviewed. BSREM identified slightly fewer lesions overall and fewer equivocal findings. ICC was excellent with regards to definitive lymph nodes and bone metastasis identification and poor with regards to equivocal metastasis irrespective of the reconstruction algorithm. The median Cohen’s kappa were 0.66, 0.74, 0.61 and 0.43 for OSEM and 0.61, 0.63, 0.66 and 0.53 for BSREM, with respect to the tumor, local lymph nodes, metastatic lymph nodes and bone metastasis detection, respectively. Conclusions: BSREM in the setting of ^68^Ga PMSA PET staging or restaging is comparable to OSEM.

## 1. Introduction

Prostate cancer is one of the most common malignancies in men. Survival is highly dependent on the stage at the time of diagnosis. Patients with tumors localized to the prostate have a better five-year survival rate than those presenting with extra-prostatic tumor growth or regional spread [1]. The finding of a single metastatic lymph node will, in many cases, change the disease stage.

The currently established methods for staging include magnetic resonance imaging (MRI) of the pelvic region, computed tomography (CT) scans of the pelvis/abdomen/thorax and bone scintigraphy [2]. An emerging method is positron emission tomography (PET) using a prostate-specific membrane antigen (PSMA)-binding radionuclide. PSMA is a type II membrane glycoprotein that is highly expressed in the prostate epithelium [3]. A low-level expression of PSMA is seen in organs such as the liver, kidneys, brain and intestines, whereas overexpression is seen in nearly all prostate cancers [4,5]. In 2012, Eder et al. laid the foundation for the ^68^Ga-labeled PSMA ligands most commonly used today [6]. Herlemann et al. showed that PSMA PET/CT was more accurate compared to CT in the nodal staging of prostate cancer, and Eiber et al. found that PSMA-PET/CT identified pathological involvement in additional regions in 24.6% of cases, compared to CT alone [7,8].

The reconstruction of detected coincidence events is needed to form the PET image. When PET was introduced in the 1970s, the analytical method of filtered-back projection was used [9]. The method was quick, but the image quality was limited by statistical noise. To account for the noise, iterative methods were developed [10]. One particularly successful iterative method called ordered subset expectation maximization (OSEM) was developed in the early 1990s by Hudson and Larkin [11]. In the past two decades, OSEM has become the clinical standard-of-care reconstruction method [12]. The algorithm cannot be run until convergence, since this leads to unacceptable levels of image noise. In particular, underestimation may occur for small lesions situated near organs with high tracer uptake, e.g., lesions close to the bladder or kidneys [13].

Recently, regularized iterative reconstruction methods have been developed. One such reconstruction algorithm is the block sequential regularized expectation maximization (BSREM) algorithm Q.Clear (GE Healthcare, Waukesha, WI, USA) [14]. BSREM introduces a penalty function to the likelihood function that provides activity dependent noise control and edge preservation at the same time as it manages to reach full convergence. A regularization parameter, β, determines the level of penalization of the relative differences between neighboring pixels. The β-value is selected at the time of reconstruction by the user, and the optimal value will typically differ depending on the radiotracer. Selecting a low β-value will result in an image with high noise and sharp contrast. Conversely, a high β-value will suppress noise but result in excessive smoothing and thereby negatively impact the resolution in the image. The differences between OSEM and BSREM reconstructed images have been studied primarily in ^18^F-Fluorodeoxyglucose (^18^F-FDG) PET imaging [15,16,17,18]. Only a few studies have been conducted with the aim to evaluate the impact of either OSEM or BSREM reconstruction on ^68^Ga-PSMA PET [19,20].

This study aims to evaluate the impact of OSEM and BSREM reconstructions in the disease staging of patients with either de novo or suspected recurrent prostate cancer imaged with whole-body ^68^Ga PSMA-11 PET/CT. Specifically, we hypothesize that (1) the BSREM reconstruction algorithm will produce fewer equivocal findings compared to OSEM reconstruction, and (2) the interrater lesion detection reliability using BSREM reconstruction will be higher compared to OSEM reconstruction.

## 2. Materials and Methods

### 2.1. Patients

OSEM and BSREM reconstructed images of patients examined with ^68^Ga PSMA-11 PET/CT were evaluated individually by three physicians. All consecutive patients, undergoing ^68^Ga PSMA-11 PET/CT for clinical reasons at the Department of Nuclear Medicine, Karolinska University Hospital, Huddinge, Sweden, between 30 August 2019 to 1 October 2020, were included in this analysis. No exclusion criteria were applied. The reasons for ^68^Ga-PSMA-11 PET/CT imaging were either baseline disease staging in biopsy-verified prostate cancer or suspected disease recurrence in patients previously treated for biopsy-verified prostate cancer. Patient age, Gleason score at time of diagnosis and plasma PSA at the time of PET/CT were recorded.

### 2.2. PET/CT Protocol

The image acquisitions were conducted using a GE Discovery MI PET/CT (GE Healthcare, Waukesha, WI, USA), digital time-of-flight (TOF) capable system, configured with four rings of detector blocks with Lutetium Yttrium Oxyorthosilicate crystals coupled to an array of silicon photomultipliers. The transaxial field of view (FOV) was 70 cm, and a 256 × 256 matrix with a voxel size of 2.73 × 2.73 × 2.79 mm^3^ was used. The PET/CT scans were conducted using 3-min acquisitions per bed position from the base of the skull to the midthigh region. Imaging was commenced with a median of 64, with a standard deviation of 6.3 min (range 52–88 min) after injection of the radiotracer. The mean administered ^68^Ga-PSMA-11 activity was 2.1, with a standard deviation of 0.14 MBq/kg (range 1.6–2.4). A low-dose CT with a tube voltage of 120 kV and a tube current of 20 mA was used for attenuation correction. A diagnostic quality neck and thoracoabdominal CT scan with intravenous contrast media under maximum inspiration was performed. Tube current modulation was applied to the diagnostic quality CT with a noise index set at 35 and a tube voltage of 120 kV. Images were reconstructed using adaptive statistical iterative reconstruction, ASiR-V, set to 50%. The imaging protocol was in accordance with the European Association of Nuclear Medicine and the Society of Nuclear Medicine and Molecular Imaging recommendations [21].

The radiotracer was compounded in our radio pharmacy department using ^68^Ge/^68^Ga generators (GalliaPharm Eckert & Ziegler Radiopharma GmbH, Berlin, Germany and GalliAd, IRE Elit Radiopharma, Fleurus, Belgium). The labeling efficiency was measured using a Scan-RAM TLC scanner with PMT detectors and software Laura for PET (LabLogic, Brandon, FL, USA). The pH of the radiopharmaceutical was controlled using pH indicator strips, and a visual inspection of the radiopharmaceutical was performed.

### 2.3. Reconstruction Algorithm

**Figure 1:**
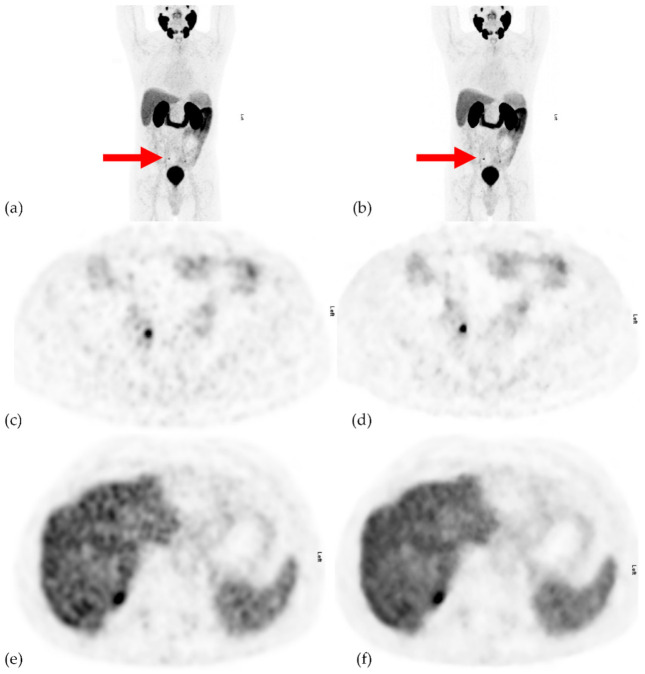
(**a**) Thick maximum intensity projection of ordered subset expectation maximization (OSEM) reconstruction. (**b**) Thick maximum intensity projection of block sequential regularized expectation maximization (BSREM) reconstruction, (**c**) transaxial OSEM reconstruction over a marked (arrow) lymph node in image **a**, (**d**) transaxial BSREM reconstruction over a marked (arrow) lymph node in image **b**, (**e**) transaxial images of the liver in OSEM reconstruction and (**f**) transaxial images of the liver in BSREM reconstruction. The lymph node in the BSREM reconstructed image in (**d**) appears more sharply defined than the OSEM reconstruction image in (**c**). The uptake of the radiotracer appears slightly smoother in the BSREM reconstruction (**f**) compared to the OSEM reconstruction (**e**).

OSEM reconstruction was performed using 3 iterations, 16 subsets and a 5.5-mm Gaussian postprocessing filter. BSREM reconstructions were performed with a β-value of 700. Both OSEM and BSREM reconstructions included the time-of-flight (TOF) and point spread function recovery and corrections for scatter, random events, dead time and CT-based attenuation correction. Figure 1 illustrates the differences between the OSEM and BSREM on a liver and a lymph node.

### 2.4. PET/CT Evaluation

**Figure 2:**
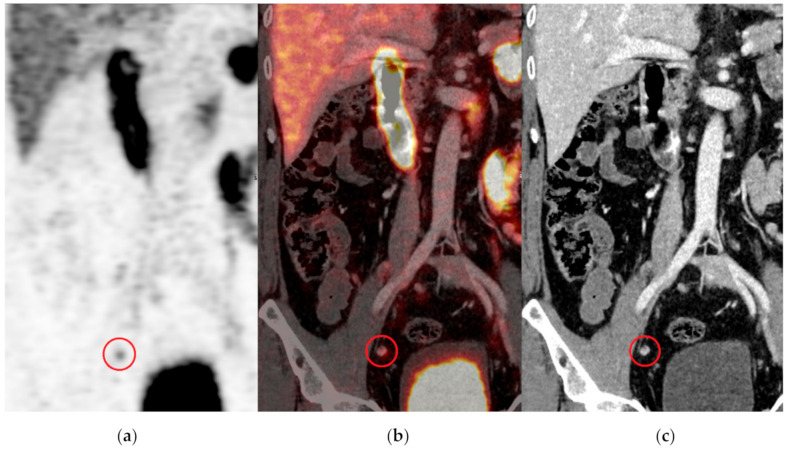
Coronal projections of (**a**) a PET, (**b**) PET-CT fusion and (**c**) CT using the OSEM reconstruction algorithm. An equivocal lymph node can be seen in the pelvis (encircled). Note the similar signal intensity of the lymph node compared to the liver uptake.

OSEM and BSREM reconstructed image series were pseudonymized individually by case numbers. Separate case numbers were used depending on the reconstruction algorithm. Reconstructed PET images were placed in individual folders, together with a matching CT image series. PET and CT images were reviewed using Hermes Hybrid viewer software (version 2.0.0, Hermes Medical Solutions AB, Stockholm, Sweden), which reconstructs image data into transaxial, coronal and sagittal images and enables comparisons with fused and un-fused PET images, as well as creates a rotating maximum intensity PET image, for overview. Two senior radiologists and nuclear medicine physicians (authors RA and SG) and an experienced radiologist (author OJ) separately reviewed the series and recorded the findings in individual databases. All findings were counted and categorized as either definitive or equivocal based on a visual comparison with the liver uptake. The findings were then grouped based on the European Association of Urology guidelines and TNM Classification of Malignant Tumors into five locales: (1) uptake consistent with local tumor/recurrences, (2) regional metastatic lymph nodes at or below the iliac artery bifurcation, (3) distant metastatic lymph nodes above the iliac artery bifurcation, (4) bone metastasis and (5) other findings clearly suspicious of prostate cancer metastasis [22,23]. Figure 2 illustrates an example of an equivocal regional lesion.

### 2.5. Statistical Analysis

All data analysis was done using IBM SPSS Statistics for Mac, version 27.0 (IBM Corp., Armonk, NY, USA). For each case, the number of findings per locale and type (definitive or equivocal) were used to calculate the intra-class correlation between the three raters. A two-way random effects model with agreement, 95% confidence limits and single measures was used. Findings were furthermore categorized for each case and locale as either 0 for no findings, 1 for only equivocal findings or 2 for at least one definitive finding. The ratings were then compared pairwise between raters per site, and Cohen’s kappa was calculated. Cohen’s kappa was also calculated to study the agreement between the OSEM and BSREM reconstructions for the three raters individually.

### 2.6. Ethics Declaration

This study was conducted in accordance with the Declaration of Helsinki. The application to the board of the Swedish Ethical Review Authority for formal ethical approval was made (application number 2018/1334-31/2). On the basis of this application, the board ruled that no ethical approval was necessary to carry out this study.

## 3. Results

Sixty-one patients were retrospectively included. See Table 1 for the patient and clinical parameters. All patients with suspected disease relapse received either primary surgical or external beam radiation therapy/brachytherapy. All but one patient in the suspected disease relapse group were referred due to changes in the prostate specific antigen (PSA). A relapse in one patient was suspected based on a highly abnormal erythrocyte sedimentation rate. The referral pattern reflects how PSMA-PET/CT was used at our department at the time of the study.

The average number of lesions per group reported by the three readers, as well as the calculated intra-class correlation coefficients (ICC), including 95% confidence limits, are presented in Table 2. The BSREM reconstructions identified fewer total lesions (175 compared to 187), fewer cases with lesions (46 compared to 50), and fewer lesions overall. The BSREM reconstructions resulted in a lower number of equivocal findings with smaller standard deviations in all locations. Intra-class agreement tests were also performed on all measurements by the two experienced raters (authors RA and SG). The results of these tests are not shown but did not yield any significant differences compared to the presented data. The number of cases with one or more lesions, depending on the reconstruction algorithm, can be found in Supplementary Table S1.

The inter-rater agreement on the lesions per groups as determined by Cohen’s kappa are presented in Table 3. The medians and ranges of the three test pairs are presented. The findings categorized as “other findings” are not reported, as the number of lesions reported was very low, with a total mean of 2.3 or 1.67 for the OSEM and BSREM, respectively. Cohen’s kappa was also used to evaluate the intra-rater agreement between the two reconstruction algorithms for each rater. The results of this analysis are presented as Supplementary Table S2.

## 4. Discussion

The methodology of our study is primarily focused on the clinical implications of using either of the two studied reconstruction algorithms by identifying the clinically relevant and easily comparable data. We chose not to mix the OSEM and BSREM cases when presenting the cases to the reviewing physicians. The smoothness of the BSREM reconstruction (see Figure 1) meant that the reviewing physicians could tell what reconstruction algorithm was used. Therefore, we prioritized avoiding that the same patient be reviewed at the same time and instead used separate folders that were reviewed at different times.

No quantitative standardized uptake value (SUV) measurements were studied. This would have been feasible but likely very time-consuming and unlikely to add any new knowledge to the question at hand. The SUV in the setting of PSMA-11 is debatable, as the uptake of a radiotracer is dependent on the receptors, which may be saturated, temporarily intracellular or nonexistent. This differs from the glucose transporter-mediated uptake of ^18^F-FDG in ^18^F-FDG PET. The signal-to-noise ratios have been extensively studied and described in a similar setting by Lindström et al. [20]. They also showed that the rated subjective agreement parameters between raters was inconsistent, and therefore, no such analysis was included in the present study.

### 4.1. Comparison of Reconstruction Algorithms

For each reconstruction algorithm, both the intra-class correlation coefficients and Cohen’s kappa were calculated. The intra-class correlation slightly favors the BSREM reconstruction with regards to the definitive lesion identification. The standard deviations are also lower within the BSREM group compared to the OSEM group (Table 2). These findings suggest BSREM may be more easily reproduced. However, for equivocal or other lesions, the intra-class correlation shows no superiority, and furthermore, the analysis of the categorized data using Cohen’s kappa (Table 3) confirms that a clinically important agreement is not consistently better for one method or the other. The agreement was, for all but bone metastasis, moderate at best. Based on our observations, the methods seem comparable. This finding is further supported by higher kappa values in the intra-rater comparisons of the two reconstruction algorithms (Supplemental Table S2) compared to inter-rater agreement (Table 3).

Fendler et al. also reported inter-rater Cohen’s kappa using OSEM-derived reconstruction algorithms [27]. For the tumor evaluation, they reported a kappa of 0.62, which is comparable with the present study with a kappa of 0.66 and 0.61 for the OSEM and BSREM, respectively. Concerning bone metastasis, Fendler et al. reported a kappa of 0.87, much higher than our study (0.43 and 0.53 for the OSEM and BSREM, respectively). Considering our intra-class correlation coefficients of definitive bone metastasis were very high, irrespective of the reconstruction algorithm, we strongly suspect that a large proportion of equivocal bone findings to be the cause of this discrepancy. Perhaps more generous criteria of the definitive bone metastasis findings would have resulted in higher kappa values. The Cohen’s kappa reported for the lymph nodes (0.74) were not comparable, as we chose to categorize lymph nodes as either regional or distant metastatic.

### 4.2. Selection of β-Value

Using ^68^Ga-PSMA PET/MR, Ter Vort et al. in 2018 found that β-values of 400 or below produced images with higher background noise compared to a reference OSEM reconstruction [19]. Meanwhile, they noted that lesions with low activity on images reconstructed with β-values above 600 had lower SUV-max compared to a reference and hypothesized that such reconstructions could possibly render small low-uptake lesions undetectable. This would suggest an optimal β-value of 400–600. At the same time, Lindström et al. found that a β-value of 900 was preferred by an experienced observer [20]. They ascribed this to the differences in sensitivity of the Signa PET/MRI and the Discovery MI PET/CT (approximately 23 versus 14 counts per seconds/kBq) and the presence of coils inside the PET FOV, resulting in higher scatter fractions. Based on these considerations, the authors suggested a β-value in the range 400–900. Our PET system had a default dual output of the OSEM and BSREM with a β-value of 700, which, considering these conclusions, seemed appropriate.

Unfortunately, the study design does not enable us to definitively verify our findings. Eiber et al. also encountered this issue in their study of recurrent prostate cancer [8]. They chose to interpret the findings as a proven positive when there was a substantial decrease in PSA following PET/CT-guided selective radiation therapy (35/248 patients). They also used follow-up imaging to verify suspicious lesions (92/248 patients). Biochemical response criteria, as suggested, seems reasonable. Follow-up imaging also seems logical in the setting of oligometastatic disease with clear partial/complete metastasis regression or clear progressing disease. Our study population was only recently examined, which means follow-up imaging is limited. Furthermore, the most challenging lesion in our study, and where the methods differ the most, were equivocal lesions in the presence of many other lesions. Based on our clinical experience, these suspected lesions are difficult to follow-up both biochemically and with imaging.

Finally, the optimal β-value may, in fact, depend on the injected dose. Messerli et al. discussed this in their study of various β-values in the setting of BSREM on ^18^F-FDG PET/CT of lung nodules. They observed “a significant shift of the selected ‘preferred image reconstruction’ towards higher β-values (i.e., from 450 to 600) in patients who received lower ^18^F-FDG doses (<2 MBq/kg) compared with patients who received higher doses (>2 MBq/kg)” [28]. In the present study, a median dose of 2.1 MBq/kg was used. Using a lower dose may thus have benefited from an even higher beta-value, and in which case, additional comparisons would have been warranted.

The BSREM reconstructions identified fewer cases with the findings: 46 versus 50 in the OSEM reconstructions. The present study lacks a follow-up or biopsy verification of the findings, and therefore, it is impossible to know which method is the most accurate in the cases with discordant findings. On the other hand, a comparison per locale showed that BSREM identifies fewer equivocal lesions overall but approximately the same number of definitive lesions. These findings suggest that BSREM reconstruction despite our otherwise ambiguous results, may be more reliably interpreted. Ter Voert et al. pointed out that BSREM with high beta-values (over 600) provide an image with low levels of noise, which could potentially explain these differences [19]. Repeat PET examinations after some time or histopathology of cases operated on would have been of great value for our analysis but outside the scope of our retrospective study. Further studies on larger patient groups and with a larger number of rating physicians would be desirable to verify if there is any benefit in using BSREM reconstruction instead of OSEM reconstruction.

## 5. Conclusions

Using the BSREM reconstruction algorithm resulted in fewer equivocal findings compared to the OSEM reconstruction and identified fewer cases with suspected recurrence. However, it remains uncertain if this has any real importance, since Cohen’s kappa on a TNM-based categorization of the data shows little difference between the two reconstruction algorithms. We concluded that BSREM reconstruction with a β-value of 700 in the setting of ^68^Ga-PMSA PET staging or restaging does not adversely impact the diagnostic accuracy and is comparable to the established OSEM reconstruction methods. In practical terms, based on our findings, the implementation of BSREM reconstruction in the clinical protocol could be considered wherever available.

## Figures and Tables

**Table 1:** Patient and tumor characteristics.

Number of patients	61
Age at exam, y, median (range)	69 (48–81)
Body mass index, kg/m^2^, median (range)	27 (20–43)
PSA pre-exam, ng/mL, median (range)	0.77 (0–788)
Gleason score, median (range)	7 (6–9)
Reason for PET/CT exam	
Suspected relapse (%)	54 (89)
De novo disease staging (%)	7 (11)
History of prior treatment	
Prostatectomy only (%)	33 (54)
Radiotherapy only (%)	14 (23)
Combined prostatectomy and radiotherapy (%)	7 (11)
Neither prostatectomy nor radiotherapy (%)	7 (11)
Time since diagnosis, years, median (range)	5 (0–20)
Median injected radiation activity MBq/kg (range)	2.1 (1.9–2.4)
Median time in minutes between injection and start of imaging (range)	64 (52–88)

**Table 2:** Mean number of lesions per group in relation to the reconstruction method and intra-class correlation coefficients calculated between all three raters for each reconstruction algorithm.

	OSEM, *n* = Lesions (SD) *	BSREM, *n* = Lesion (SD) *	OSEM ICC (95% CI)	BSREM ICC (95% CI)
Local uptake, definitive	14.3 (1.2)	13.7 (3.2)	0.61 (0.48–0.73)	0.68 (0.56–0.78)
Regional lymph nodes, definitive	45 (6.6)	47 (2.1)	0.73 (0.62–0.82)	0.91 (0.86–0.94) ^†^
Metastatic lymph nodes, definitive	75 (12)	76 (5)	0.97 (0.95–0.98) ^††^	0.98 (0.97–0.99) ^††^
Bone metastasis, definitive	30 (2.5)	34 (2.6)	0.98 (0.97–0.99) ^††^	0.98 (0.97–0.99) ^††^
Local uptake, equivocal	6.7 (3.5)	6 (3)	0.32 (0.17–0.49)	0.10 (−0.05–0.27)
Regional lymph nodes, equivocal	20 (10)	13 (4.5)	0.42 (0.26–0.57)	0.15 (−0.004–0.32)
Metastatic lymph nodes, equivocal	19 (9.8)	13 (2.5)	0.30 (0.14–0.46)	0.34 (0.18–0.50)
Bone metastasis, equivocal	20 (16)	13 (14)	0.52 (0.33–0.67)	0.20 (0.05–0.36)
Other findings clearly suspicious of prostate cancer	2.3 (2.1)	1.67 (1.5)	0.19 (−0.07–0.42)	0.27 (0.02–0.49)
Total reported lesions	187 (27)	175 (22)		
Cases with any findings	50 (4.5)	46 (3.5)		

* Total number of lesions reported, mean of the tree raters (standard deviation). ^†, ††^ Based on the suggestions of Koo et al. [24] and Liljequist et al. [25], the intra class-correlation are classified as moderately reliable if the values are between 0.50 and 0.75, good when the lower limit of the 95% confidence interval (Cl) is between 0.75 and 0.90, marked ^†^, and excellent intra-class correlation (ICC) when the entire 95% CI is above 0.90, marked ^††^. OSEM: ordered subset expectation maximization and BSREM: block sequential regularized expectation maximization.

**Table 3:** The inter-rater agreement of the lesions per group findings depending on the reconstruction algorithm.

	OSEM		BSREM	
	Cohen’s Kappa *	Range ^†^	Cohen’s Kappa *	Range ^†^
Local tumor	0.66	0.53–0.73	0.61	0.54–0.65
Regional lymph nodes	0.74	0.72–0.77	0.63	0.62–0.74
Metastatic lymph nodes	0.61	0.55–0.65	0.66	0.60–0.69
Bone metastasis	0.43	0.37–0.72	0.53	0.53–0.78

* Median of paired tests between all raters. Interpretation of Cohen’s kappa as recommended by McHugh [26]: below 0.60 = weak at best, 0.60–0.79 = moderate, 0.80–0.90 = strong and above 0.90 = almost perfect. ^†^ Range of the three paired tests.

## Data Availability

The analyzed data, including case ratings, may be obtained from the authors upon request. PET and CT datasets cannot be shared due to ethical and practical reasons.

## Supplementary Materials

The following are available online at https://www.mdpi.com/article/10.3390/diagnostics11040630/s1: Table S1: Mean number of cases with lesions in relation to the reconstruction method and Table S2: Intra-class correlation comparisons of OSEM and BSREM using Cohen’s kappa.
