# Supplementary material for: Comparison of Regularized Reconstruction and Ordered Subset Expectation Maximization Reconstruction in the Diagnostics of Prostate Cancer Using Digital Time-of-Flight 68Ga-PSMA-11 PET/CT Imaging
Source: Diagnostics (Basel). 2021 Mar 31;11(4):630. doi: 10.3390/diagnostics11040630 (PMC8067147; doi:10.3390/diagnostics11040630)
Supplement: Supplementary file 1 [file diagnostics-11-00630-s001.pdf]

diagnostics

MDPI

Table S1 Mean number of cases with lesions in relation to reconstruction method.

	OSEM, n=cases with lesions (SD)	BSREM, n=cases with lesions (SD)
Local uptake, definitive	12.3 (2.1)	12.3 (2.1)
Regional lymph nodes, definitive	19.3 (3.2)	22 (2)
Metastatic lymph nodes, definitive	10.7 (2.9)	12 (2)
Bone metastasis, definitive	14.3 (1.5)	15.3 (1.5)
Local uptake, equivocal	5.3 (2.5)	5 (2)
Regional lymph nodes, equivocal	14.3 (6.1)	9.7 (3.2)
Metastatic lymph nodes, equivocal	10.7 (4.0)	7 (0)
Bone metastasis, equivocal	14.6 (11.5)	9.7 (9.9)
Other findings clearly suspicious of prostate cancer	2 (2)	1.3 (1.2)

Table S2 Intra-class correlation comparisons of OSEM and BSREM using Cohen's kappa.

	Rater OJ	Rater RA	Rater SG	Mean
Local tumour	0.79	0.73	0.80	0.77
Regional lymph nodes	0.81	0.71	0.60	0.71
Metastatic lymph nodes	0.80	0.74	0.65	0.73
Bone metastasis	0.75	0.74	0.64	0.71

Interpretation of Cohen's kappa as recommended by McHugh [26]: below 0.60 = weak at best, 0.60-0.79 = moderate, 0.80-0.90 = strong, above 0.90 = almost perfect.
